# Sofosbuvir plus Ribavirin is effective for HCV elimination in people living with HIV from rural area of China

**DOI:** 10.1038/s41598-021-90706-5

**Published:** 2021-05-28

**Authors:** Liyu Chen, Lingyao Du, Shuang Kang, Fanghua Ma, Changmin Li, Min He, Lang Bai, Hong Tang

**Affiliations:** 1 Center of Infectious Diseases, West China Hospital of Sichuan University, Chengdu, 610041 China; 2 Center of Antiretroviral treatment, People’s Hospital, Zhaojue County, Liangshan Yi Autonomous Prefecture, 616150 China

**Keywords:** Hepatitis C virus, Retrovirus

## Abstract

People living with HIV (PLWH) bear higher prevalence of HCV coinfection. An accessible directly acting antivirals regimen with less drug–drug interaction with antiretroviral therapy (ART) is urgently needed in source limited regions. We aimed to assess the efficacy and safety of SOF + RBV for 24 weeks regimen in HIV–HCV coinfected patients in Liangshan Prefecture, China. PLWH under ART from China’s national free antiretroviral treatment project (CNFATP) and diagnosed with treatment-naïve HCV infection were enrolled. SOF + RBV was administrated for 24 weeks and patients were followed for ≥ 12 weeks. The efficacy and safety were analyzed and related factors were explored. 58 patients completed 24 weeks of SOF + RBV and had all tests done. Genotype prevalence in this population was G3 44.8% (n = 26), G6 31.0% (n = 18) and G1 17.2% (n = 10) respectively. 52/58 (89.7%) patients achieved SVR12 while 10.3% experienced therapeutic failure. However, SVR12 was neither significantly different between groups of different gender, age, transmission routines, CD4^+^ cell count, HIV infection duration, ART duration and HBsAg prevalence nor influenced by HCV viral load, genotypes and hepatic stiffness. The regimen was well-tolerated without any serious AEs or AEs leading to treatment adjustment or discontinuation reported. PLWH in Liangshan showed a high prevalence of HCV coinfection with GT3 and GT6 as the most frequent genotypes. SOF + RBV for 24 weeks could achieve good SVR12 in this population and was well-tolerated. It has great potential to be generalized in coinfected population in source-limited regions.

## Introduction

People living with human immune-deficiency virus (PLWH) are known to bear higher risk of hepatitis C virus (HCV) infection because of shared transmission routes and risk factors. Globally, HCV prevalence is 6.2% (IQR 3.4–11.9) among the 38 million PLWH, 5.8 times of that in general population^1^. The coinfection is even more severe in the subpopulation of men who have sex with men (MSM) with an estimated prevalence of 6.4% and people who inject drugs (PWID), 82.4%^1^. Coinfection with HIV leads to more dysregulated immune system, lower chance of spontaneous control of HCV infection and faster progression of liver disease than HCV mono-infection^2^ while coinfection with HCV is associated with suboptimal immune reconstitution^3, 4^ and increased viral reservoir size^5^ compared with HIV mono-infection. Thus, engagement of coinfected patients in HCV treatment is undoubtfully an important aspect of public health issues.

The advent and development of direct-acting antivirals (DAAs) has changed the paradigm of HCV treatment. Target of HCV infection management is sustained viral response (SVR) defined as undetectable HCV-RNA 12 or 24 weeks after completing antiviral therapy, which usually signifies the cure of HCV except for rare relapse. Currently, DAAs can achieve an SVR rate upwards of 90% in HCV mono-infected patients^6, 7^. In May 2016, the World Health Assembly endorsed the Global Health Sector Strategy (GHSS) on viral hepatitis, which calls for the elimination of viral hepatitis as a public health threat by 2030 (reducing new infections by 90% and mortality by 65%)^8^. With the high prevalence and re-infection risk, HCV infection in PLWH is considered the priority on the way to realize hepatitis elimination. Multiple trials and real-world studies have been carried out to investigate the efficacy and safety of DAAs in HIV–HCV coinfected patients. DAAs are demonstrated to result in SVR rates comparable to that in HCV mono-infection settings^9^ and real-world evidence further corroborated these findings^10, 11^. Therefore, treatment against active HCV infection defined as detection of HCV RNA is now generally recommended for all HCV patients regardless of HIV infection status. Although in coinfected patients, extra cautions are warranted to avoid drug-drug interactions (DDIs) and consideration might be required on the timing of DAA initiation.

Evidences from global observations have revealed China as one of the countries with the highest burden of HCV in PWID^1, 12^. Liangshan Prefecture is one of the areas with the highest HIV prevalence in China. This remote, impoverished district is domiciled mostly with ethnic minority people of Yi nationality who account for 88.07% of new HIV infections from 2011 to 2013 (4). With the dominant transmission routes of injecting drug use and heterosexual behaviors, 45.7–61.9% of PLWH in Liangshan Prefecture are found to be positive for anti-HCV antibody in previous studies conducted by us and others^3, 13^. PLWH with unique characteristics in this region have always been a major concern and the issue of HCV coinfection is a key part. Since 2020, three DAAs are included in Chinese National Reimbursement Drug List, allowing the cost of HCV treatment accessible for average patients. However, PLWH in Liangshan is the exception with DAAs still far beyond reach due to economic considerations, despite the massive demand. Moreover, as almost all patient in this area is now under China’s national free antiretroviral treatment program (CNFATP) consisting of nucleoside reverse transcriptase inhibitors (NRTIs) plus non-nucleoside reverse transcriptase inhibitors (NNRTIs) or protease inhibitors (PIs), tentatively switching to integrase inhibitor (INSTIs) to minimize the DDIs is also unaffordable for them. Thus, an accessible DAA regimen with less DDI with NRTIs, NRTIs and PIs is urgently need.

Besides, considering the special HIV epidemics in this area, the molecular epidemics of HCV might also be different and require specific treatment considerations. However, there is scanty observation of genotype (GT) distribution as well as efficacy evaluation of DAA in this peculiar population in Liangshan. Studies from other regions reported genotype 3 HCV strain always took considerable portion in similar coinfected patients^14, 15^. The pan-genotypic regimen is recommended in such patients if genotype sequencing is not accessible. Sofosbuvir (SOF) plus ribavirin (RBV) for 24 weeks, the original interferon-free pan-genotypic regimen with only one NS5B inhibitor, has the advantages of cost-effectiveness, accessibility and less DDIs. As the SVR is still over 90% in treatment-naïve patients and around 70% in treatment experienced patients^16^. This regimen might have great potential to be applied in coinfected population in source-limited regions. In the coming years, generic sofosbuvir would be issued in Chinese pharmaceutical market^17^. It would largely increase the accessibility of the SOF + RBV regimen in HIV–HCV coinfected patients from rural regions, even facilitating to establish a new national management strategy for them if the efficacy and safety was confirmed.

To address all these issues, we conducted a prospective study to assess the efficacy and safety of SOF + RBV for 24 weeks regimen in HIV–HCV coinfected patients in Liangshan Prefecture.

## Methods and materials

### Study aim and design

This is a prospective cohort study aimed to assess the efficacy and safety of an accessible interferon-free DAA-containing regimens “SOF + RBV for 24 weeks” in HIV–HCV co-infected patients from Liangshan Prefecture, a rural area of China.

### Subject enrollment

HIV patients receiving antiretroviral therapy (ART) from CNFATP were screened in May, 2019. Those with positive anti-HCV antibody were further detected for HCV RNA. Inclusion criteria included patients with confirmed HIV infection, ART duration > 1 year, HIV viral load < 1000 copies/mL, and HCV viremia. Patients were excluded who were pregnant or breastfeeding, with platelet counts < 20 × 10^9^/L, with estimated glomerular filtration rate (eGFR) < 30 mL/min per 1.73 m^2^, with advert or unstable cardiac diseases, with active opportunistic infections, with history of previous anti-HCV treatment and with no or limited disposing capacity which would hamper medication compliance.

### Study procedure

In consideration of the medical cost and drug availability when generalizing the regimen in this region, we chose to use SOF (400 mg once daily, [QD]) + RBV (1000 mg QD for those < 75 kg or 600 mg twice a day for those ≥ 75 kg) for 24 weeks based on the guidelines for HCV treatment^18^. Patients were all receiving ART based on 2 NRTIs (tenofovir [TDF], lamivudine [3TC], zidovudine [AZT]) + 1NNRTI (nevirapine [NVP], efavirenz [EFV]) or PI (lopinavir/ritonavir [LPV/r]). Considering potential DDIs between anti-HIV and anti-HCV agents as well as drug availability, AZT was changed to TDF and NVP was changed to EFV.

At baseline, sociodemographic variables, medical history and medical profile of HIV infection status were collected from the national database. Physical examinations, HCV RNA, HCV genotypes, laboratory tests including hematology, liver and renal function and hepatic ultrasound evaluation were performed. Non-invasive methods of serum biomarker panels were reported to be less accurate for liver fibrosis and cirrhosis assessment in HIV–HCV coinfected patents^19, 20^, but transient elastography was not accessible in this region. Instead, we adopted aspartate aminotransferase (AST)-to-platelet index (APRI) in collaboration with fibrosis 4 (FIB-4) scores to assess livers stiffness with higher sensitivity and specificity^21, 22^. Advanced liver fibrosis, which would easily progress to cirrhosis, and liver cirrhosis was considered when patients had an APRI score above 1.0 and a FIB-4 score above 3.25. In our study, we refer to them with “hepatic cirrhosis” for convenience. The threshold of ARPI was set at 1.0 according to a meta analysis from 40 studies. The researchers concluded that an APRI score over 1.0 had a 76% sensitivity and 72% specificity for predicting cirrhosis. However, an APRI cut-off of 2.0 was more specific (91%) but less sensitive (46%)^23^. Therefore, considering both the sensitivity and specificity, the threshold value of APRI for the diagnosis of cirrhosis was selected as 1.0 in our study. The threshold of FIB-4 for the diagnosis of cirrhosis is slightly different in different liver diseases. Vallet-Pichard et al. proved that FIB-4 is an inexpensive and accurate non-invasive marker of cirrhosis in patients with HIV/HCV coinfection in their study. They found most patients with FIB-4 < 1.45 had no obvious liver fibrosis or only moderate liver fibrosis below grade 2, and the coincidence rate with liver biopsy was 94.7%. Patients with FIB-4 index > 3.25 had advanced liver fibrosis of grade 3–4 or cirrhosis, with the coincidence rate of 82.1% to liver biopsy^24^. Therefore, we chose the FIB-4 index > 3.25 as the threshold in our HIV/HCV co-infected patients.

Patients enrolled were then administered with sofosbuvir plus ribavirin for 24 weeks and followed up at Week 4 and 12. HCV RNA was measured at each visit. Laboratory tests were repeated when HCV therapy was completed. Adverse events were recorded and all adverse events grade ≥ 3 or any grade that led to treatment discontinuation were reported. Virologic failure for HIV infection was defined as an increase in HIV RNA to ≥ 1000 copies/mL at any time after study entry. HCV virologic failure was defined: (1) The failure to achieve HCV RNA < lower limit of quantification (LLOQ) by treatment Week 24 or (2) An HCV RNA rebounding to ≥ LLOQ after achieving undetectable HCV RNA at any time during the study.

The efficacy was determined as the percent of patients achieving SVR at 12 weeks (SVR12) after completion of HCV therapy. Secondary outcomes included SVR12 by HCV genotype, SVR12 by baseline CD4^+^ count levels, changes of HIV viral load, changes of CD4^+^ counts and adverse events. Factors associated with SVR were also explored.

### Statistics

Category variables were described as number and percent and analyzed with chi-square test. Continuous variables were described as median and interquartile range (IQR) or mean and standard deviation (SD) and analyzed by *U* test or *t* test as appropriate. Multivariate logistic regression was used to determine whether there were any correlations between SVR and those independent variables with significant differences (*P* < 0.05) and of clinical interest. The predictive variables included were defined a priori and included gender, age, HCV genotype, presence of cirrhosis, and HCV RNA. Odds ratios (ORs) and *P *values are presented where applicable. The significance level established for all analyses was 0.05.

### Ethics approval and consent to participate

This study was approved by the Medical Ethics Committee of West China Hospital of Sichuan University and written informed consents were acquired from all participants (Annual Audit No. 450, Version 2019.5). All methods were performed in accordance with the relevant guidelines and regulations.

## Results

### Patient demographics and baseline clinical characteristics

A total of 58 patients completed 24 weeks of DAA-containing therapy and had all tests done. The majority (50/58, 86.2%) were males at their mid ages (40 [35, 44] years old). Intravenous drug use (IDU) was the most frequent transmission route for HIV and HCV acquisition and was present in 98.3% (57/58) of patients. The prevalence of triple infection of HIV/HBV/HCV was 5.2%. The median duration of HIV acquisition was 8.0 (5.0, 11.0) years. After a median of 6.0 (2.0, 7.0)-year ART, all patients achieved virological response (HIV viral load < 1000 copies/mL) with 79.3% reaching complete viral suppression (HIV viral load < 50 copies/mL) while the recovery of CD4^+^ counts was less satisfactory with 51.7% of patients demonstrating a CD4^+^ count < 350 cells/μL. To be exact, there were 13 patients with very in-satisfactory immune systems of CD4^+^ counts < 200 cells/μL, 17 patients with in-satisfactory immune systems of 200 ≤ CD4^+^ counts < 350 cells/μL, 12 patients with moderately satisfactory immune systems of 350 ≤ CD4^+^ counts < 500 cells/μL while 16 patients with full satisfactory recovery of CD4^+^ counts ≥ 500 cells/μL (Table 1).

**Table 1:** Baseline characteristics other than HCV infection of study subjects (grouped by SVR12).

	N = 58	SVR12 (N = 52)	Non-SVR12 (N = 6)	*P* (SVR12 vs. non-SVR12)
**Gender (cases, ratio)**				0.829
Male	50 (86.2%)	45 (86.5%)	5 (83.3%)	
Female	8 (13.8%)	7 (12.1%)	1 (1.7%)	
**Age (years)**	40.0 (35.0, 44.0)	40.0 (36.0, 44.0)	38.5 (32.0, 46.0)	0.673
**Routes of infection**				0.732
IDU	57 (98.3%)	51 (98.1%)	6 (100%)	
Heterosexual contact	1 (1.7%)	1 (1.9%)	0	
**Baseline CD4**^**+**^** count (cells/μL)**	378 ± 189	358 (200, 543)	328 (279, 363)	0.619
**Baseline CD4**^**+**^ **count (cells/μL) grade**				
< 200	13 (22.4%)	13 (25.0%)	0	
200 ≤ CD4^+^ < 500	29 (50.0%)	23 (44.2%)	6 (100%%)	
≥ 500	16 (27.6%)	16 (30.8%)	0	
**HIV Infection duration (years)**	8.0 (5.0, 11.0)	7.5 (5.3, 11.0)	10.0 (4.0, 11.0)	0.605
**ART duration (years)**	6 (2, 7)	6 (2, 7)	4 (1.8, 7.3)	0.503
**HBsAg positive**	3 (5.2%)	2 (3.8%)	1 (16.7%)	0.179

As for HCV characterization, most patients were with prolonged HCV infection and HCV RNA levels were consistently high (6.31 [5.50, 6.68] log10 IU/mL) across subgroups of different HCV genotypes. Genotype prevalence found in this population was as follows: G3 44.8% (n = 26); G6 31.0% (n = 18); G1 17.2% (n = 10). All the patients demonstrated basically normal liver function parameters. As determined by APRI and FIB-4 scores, 17.2% of patients were with liver cirrhosis at baseline. Among them, 5 patients were with GT3 HCV infection including 2 with GT3a and 3 with GT3b. Only 1 cirrhotic patient was with GT1b HCV infection and the rest 4 patients were with GT6xa HCV infection. There was no significant difference of liver cirrhosis prevalence between different genotype subgroups.

### Effectiveness and safety

**Figure 1:**
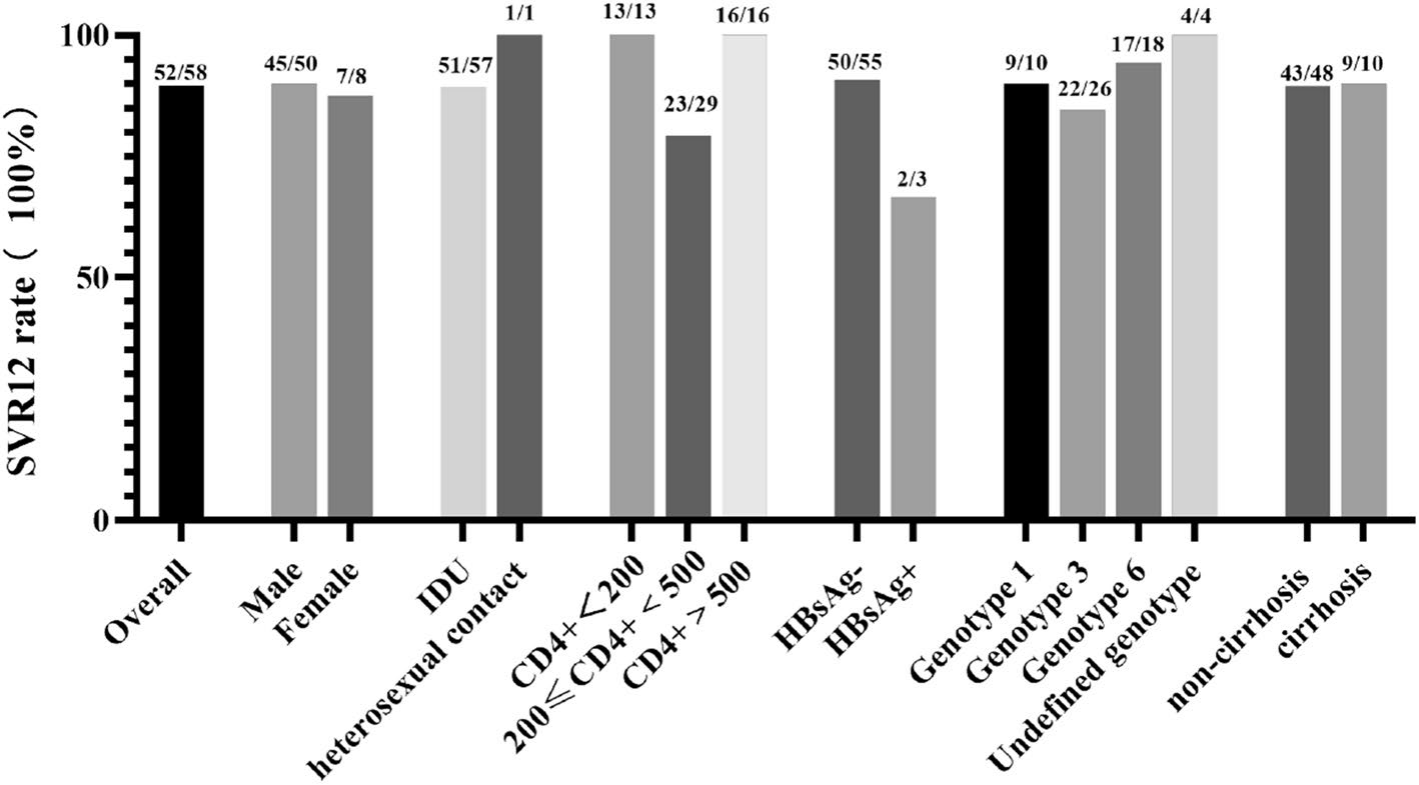
SVR12 rate in HIV–HCV coinfected patients of different subgroups.

Among the patients with all duration completed and all test done, 50/58 (86.2%) patients achieved rapid viral response defined as HCV RNA being undetectable after 4 weeks of treatment. Afterwards, 5 more patients achieved undetectable HCV RNA but 3 patients with RVR suffered virologic relapse at end of treatment (EOT, Week 24). Overall, 52/58 (89.7%) patients reached SVR12 while 10.3% experienced therapeutic failure. The SVR12 rate in each subgroup divided by mentioned parameters ranging from 66.67% (2/3) in patients with triple infections to 100% (Fig. 1).

However, SVR12 was neither significantly different between groups of different gender, age, transmission routines, CD4^+^ cell count, HIV infection duration, ART duration and HBsAg prevalence (Table 1) nor influenced by HCV viral load, genotypes and hepatic stiffness (Table 2). During the regression analysis, no positive risk factors were identified to influence the SVR12 either.

**Table 2:** Baseline HCV characterization of study subjects (Grouped by SVR12).

	N = 58	SVR12 (N = 52)	Non-SVR12 (N = 6)	*P* (SVR12 vs. non-SVR12)
**HCV Infection duration (years)**	3.0 (2.0, 4.3)	3.0 (2.0, 4.8)	2.0 (1.0, 3.5)	0.100
**HCV RNA (log** _10_** IU/mL)**	6.3 (5.5, 6.7)	6.2 (5.2, 6.6)	6.8 (6.1, 7.3)	0.059
**HCV genotype**				0.590
3	26 (44.8%)	22 (42.3%)	4 (66.7%)	
3b	16 (27.6%)	14 (26.9%)	2 (33.3%)	
3a	10 (17.2%)	8 (15.4%)	2 (33.3%)	
6	18 (31.0%)	17 (32.7%)	1 (16.7%)	
6xa	12 (20.7%)	12 (23.1%)	0	
6a	3 (5.2%)	3 (5.8%)	0	
6n	3 (5.2%)	2 (3.8%)	1 (16.7%)	
1	10 (17.2%)	9 (17.3%)	1 (16.7%)	
1b	7 (12.1%)	7 (13.5%)	0	
1a	3 (5.2%)	2 (3.8%)	1 (16.7%)	
Undefined	4 (6.9%)	4 (7.7%)	0	
**Hepatic cirrhosis**	10 (17.2%)	9 (17.3%)	1 (16.7%)	0.969

Six patients with treatment failure were all with HIV viral loads < 1000 copies/mL and the median CD4^+^ cell count was 328 (300, 348) cells/μL. HCV RNA of these patients tended to be slightly higher than that of those achieving SVR12 though no significant difference was observed (6.56 log10 vs. 6.31 log10, *p* = 0.059). Three patients experienced relapse with HCV RNA rebounding from undetectable at EOT to detectable 12 weeks after treatment and were all infected with GT3 HCV (1 with GT3a and 2 with GT3b). The other three patients never achieved undetectable HCV RNA during the study and were infected with GT1a, 3a, 6n HCV separately (one patient each). Further analysis did not identify any significant predicting factor of treatment failure, possibly attributed to the limited sample size (especially that of the treatment failure group) and relatively homogenous study population.

SOF + RBV for 24 weeks was well-tolerated with no serious AEs (grade ≥ 3) or AEs leading to treatment adjustment or discontinuation reported. The most common AEs included nausea, vomiting, abdominal discomfort, and fatigue. Three patients were found with anemia (decreased hemoglobin levels of < 10 g/dL) due to ribavirin at EOT, but were all reversible during the subsequent follow-up period. The incidence of all recorded adverse events was not significantly different between responders and non-responders (details in Table 3). All patients maintained sustained HIV virologic response under ART. There was no liver enzyme level increasing to > 5 times upper limit of normal during the treatment and the liver enzyme were comparable between patients with SVR12 or not.

**Table 3:** Adverse events in study subjects (grouped by SVR12).

Adverse events	N = 58	SVR12 (N = 52)	Non-SVR12 (N = 6)	*P* (SVR12 vs. non-SVR12)
Nausea	1 (1.7%)	0	1 (16.7%)	0.103
Vomiting	5 (8.6%)	5 (9.6%)	0	1.000
Abdominal discomfort	14 (24.1%)	12 (23.1%)	2 (33.3%)	0.624
Fatigue	36 (62.1%)	33 (63.5%)	3 (50.0%)	1.000
Anemia	3 (5.17%)	3 (5.76%)	0	1.000

## Discussion

With the striking HIV prevalence and previously rampant illicit drug deals, disease burden of chronic hepatitis C is quite severe and requires prompt and efficient management in Liangshan Prefecture. The prevalence of anti-HCV antibody in PLWH is around 50% across several cross-sectional studies and our investigation further revealed GT3 and GT6 as the predominantly circulating genotypes, which is consistent with previous findings about the HCV epidemics in southern China^25^. GT3 has long been associated with faster progression of fibrosis and a greater risk for HCC and all-cause mortality than other HCV genotypes^26, 27^ and thus is considered hard-to-treat, making HCV eradication even more urgent a need in our study population.

Though the burden of HIV–HCV in Liangshan Prefecture is heavy, evidence concerning the efficacy and safety of DAA-based treatment there has been scarce. As all patients there could only access CNFATP, DDI between DAAs and ART should be further concerned to prevent either the toxicity from up-regulated drug exposure or the treatment failure from down-regulated drug concentration^28^. At current price, the genotype-dependent regimen is cost-saving compared to pan-genotypic regimen if the HCV genotypic sequencing is convenient and genotype 1 or 4 has been determined. Otherwise, pan-genotypic regimen is recommended^29^. Studies on the peculiar HCV genotypic distribution in minority regions reported considerably high portion (over 70%) of non-genotype 1 or 4 strains in similar cohort^30, 31^. As mentioned before, we have shown that PLWH coinfected with mostly GT3 (44.8%) and GT6 (31%) HCV in Liangshan. Along with limited accessibility to genotypic sequencing, it makes the pan-genotypic regimen more recommended there.

Velpatasvir-based pan-genotypic regimen (commonly sofosbuvir plus velpatasvir, SOF/VEL) is cost-effective for HCV treatment in India where generic drugs are available but not in China^29^. The price of original SOF/VEL makes it hard to afford in Chinese minority regions. Likewise, INSTIs were not easily affordable to minimize the DDI between velpatasvir and ART. As a result, the SOF + RBV for 24 weeks, a cost-saving pan-genotypic regimen with only one DAA, has emerged to be an optimal candidate regimen for coinfected patients. We conducted a pilot study to assess the efficacy and safety of SOF + RBV for 24 weeks in HIV–HCV coinfected patients in Liangshan Yi nationality Prefecture. In a meta-analysis of 33 studies across 16 countries, the estimated SVR rate in 640 HIV–HCV co-infected patients treated with SOF + RBV was 80% (95% CI 73–87%) with an AE incidence > 5%^32^. Our study proved that SOF + RBV for 24 weeks could achieve acceptable SVR rate of around 90% in HIV–HCV coinfected patients in Liangshan area, providing evidence of applicable HCV treatment options. The SVR12 in this population is higher than the previous studies. It might partially result from our strictly monitoring during the treatment. In Liangshan, every HIV infected patient will receive two kinds of monitoring: the regular on-site visit to physicians in the county hospital and a daily face-to-face supervision on his/her cART adherence from a village doctor in his residence. During the study period of 6 month’s medication days, the adherence of DAAs was strictly supervised by these rural doctors as well. The medication adherence was ensured to 100% in each patient unless the treatment had been modified. Most importantly, all our study subjects were treatment-naïve and accepted ribavirin with full dosage and course. Similar high SVR12 was reported in previous treatment-naïve cohort^16^.

Benefits from anti-HCV treatment in this population is expectable and could be realized. Moreover, the meta-analysis showed that SOF/DCV ± RBV would be the priority for GT3 patients with the excellent SVR rates of 97% and that SOF/VEL and SOF/LDV ± RBV could serve as alternatives with SVR rates above 90%^32, 33^. These regimens should be considered for generalized anti-HCV treatment in the future but the incidence of DDI should also be monitored. Besides, cure of HCV was reported to be associated with multiple benefits in coinfected patients, including decreased markers of chronic immune activation, microbial translocation, shrunken reservoir size, and improved cognition^5, 34^. Long-term follow-up of these treated patients might bring more evidence for improved patient management.

To our best knowledge, this is the first investigation looking into the HCV landscape among this specific population. It demonstrated the regimen was with an SVR12 of approximately 90% and was well tolerated, comparable to the report that SVR of SOF + RBV for 24 weeks is still over 90% in treatment-naïve patients and around 70% in treatment experienced patients^16^. Ribavirin contained-regimens might have more mild or moderate adverse events than those without. But our cohort did not show any severe ones result in treatment discontinuation or therapeutic failure. This regimen might have great potential to be generalized in coinfected population in source-limited regions.

There are certain limitations of this study. The sample size was relatively small and almost all patients were IDU, rendering our findings less representative of patients infected through sexual contact. Determination of hepatic cirrhosis was based on APRI and FIB-4 scores, and the thresholds of defining cirrhosis were set to include a few patients with advanced liver fibrosis, which might not be adequate. The resistance-associated substitutions were not detected due to extra financial burden for patients in non-SVR12 group. Based on experiences from this study, elaborately-designed trial with larger sample is expected to provide more evidence to guide better management of HCV in PLWH in rural regions like Liangshan Prefecture.

## Conclusions

PLWH in Liangshan Prefecture showed a high prevalence of HCV coinfection with GT3 and GT6 as the most frequent genotypes. SOF + RBV for 24 weeks could achieve good SVR in this population and was well tolerated. It has great potential to be generalized in coinfected population in source-limited regions. Studies in larger population are needed to vindicate these findings.

## Data Availability

The data used and/or analysed during the current study are available from the corresponding author on reasonable request.
